# Exploring challenges among Iranian family caregivers of seniors with multiple chronic conditions: a qualitative research study

**DOI:** 10.1186/s12877-022-02881-3

**Published:** 2022-04-01

**Authors:** Sahar Malmir, Hassan Navipour, Reza Negarandeh

**Affiliations:** 1 Nursing Department, Faculty of Medical Science, Tarbiat Modares University, Tehran, Iran; 2 Department of Nursing, School of Medical Sciences, Tarbiat Modares University, Tehran, Iran; 3 Nursing and Midwifery Care Research Center, School of Nursing and Midwifery, Tehran University of Medical Sciences, Tehran, Iran

**Keywords:** Family Caregiver, Seniors with Multiple Chronic Illness, Challenges, Qualitative Research, Content Analysis

## Abstract

**Background:**

It is believed that seniors with multiple chronic diseases are in dire need of support from their family caregivers; however, it can impose a significant burden on these caregivers. Therefore, it is imperative to take into account caregivers’ needs, as covert patients, along with the needs of patients; besides, it is necessary to develop supportive and health promotion programs for them. There is a critical gap in the knowledge about health problems related to family caregivers of the growing population of these senior citizens. The present study aimed to explain the challenges imposed on family caregivers of seniors with several chronic diseases in Iran.

**Methods:**

This study was conducted based on the conventional qualitative content analysis method. For this purpose, 13 family caregivers of seniors with several chronic diseases were selected using the purposive sampling method. The study population included those referred to two health centers and outpatient clinics of two public hospitals in Khorramabad, Iran. Data were collected through semi-structured interviews. Data analysis was performed along with data collection using inductive thematic analysis proposed by Elo and Kyngäs. Besides, Guba and Lincoln**’s** criteria were used to ensure the trustworthiness of the data.

**Results:**

The analysis of the obtained data led to the identification of challenges of family caregivers of the older patients with multiple chronic diseases; these challenges were classified into six main categories, including the impact of caregiving on family relationships, disruption of social relationships, disruption of personal and occupational plans, physical health-related issues, negative emotions, and dealing with the high costs of care.

**Conclusions:**

Given that family caregivers may face several challenges while taking care of seniors with multiple chronic diseases, healthcare providers should design and plan various interventions based on such challenges using **a** caregiver-centered approach.

## Background

The global seniors’ population is estimated to reach two billion people by 2050 [1]. It is predicted that the population older than 60 years will constitute more than 10% and 20% of the Iranian population in 2021 and 2050, respectively [2]. It is noteworthy that the risk of developing one or more chronic diseases increases with aging [3]. Multiple chronic conditions (MCCs) refer to two or more chronic diseases that have increased among adults in the last 20 years worldwide [4], and 55% to 98% of the seniors are reported to have two or more chronic diseases [5]. Seniors with MCCs may face challenges in self-care interventions [3]. These people often suffer from physical and mental problems that lead to further complexity in providing care [6]. Such senior residents are more likely to experience medical and pharmaceutical complications [7]. Furthermore, there will be numerous adverse consequences due to frequent MCCs, for instance, higher reference to healthcare providers, lower quality of life, as well as more frequent hospitalization and even morbidity [4]. Finally, having MCCs can impair patients' ability to adhere to treatment and conduct self-care interventions, which increases their dependence on family caregivers and the likelihood of adverse health outcomes [7, 8].

Family caregivers can only provide up to 80% of the required care to seniors with MCCs in the community, and they are also responsible for the majority of the costs and shoulder the related burden [4, 8]. The existing research evidence shows that the seniors usually prefer to receive care at home, which is in line with the government's plan to hand over caregiving responsibilities to families at home [8]. As a result, the formal medical duty has shifted from caregiving experts such as nurses and physicians to family caregivers. Family caregivers take responsibility for most of the care tasks (from personal care to medical care) to seniors with MCCs in the community; besides, complimentary services are provided for 1–2 h per week by the health care system. Consequently, caregiver strain is a growing concern [8]. There are various pressures on caregivers, such as helping patients in daily life activities, managing multiple appointments with health care professionals in different locations, and helping patients adhere to multiple and complicated medication regimens. Such a heavy burden may put much pressure on caregivers [9]. Caregivers for seniors with MCCs may experience a significant caregiving burden due to changes in their roles and responsibilities [4]. Caregivers’ burden refers to "the ongoing impact of providing care for a dependent, seniors, relative with a disability or some type of deterioration" that threatens the caregiver's physical and mental health, family relationships, occupation, and financial status [10]. Family caregivers experience overwhelming pressure by providing care to seniors with MCCs and feel undervalued and under-supported by the health care system [4]. Therefore, such caregivers may not be able to continue their role. If no other family members or friends can take responsibility for care, the seniors may need to be admitted to a long-term care center or nursing home, which will incur high costs on medical systems [11].

As a family-oriented society with strong traditional conventions, most of Iranians believe that it is heavenly to support and take care of the older members of the family at home [12]. Nonetheless, some studies investigated the home caregiving conditions and concluded that the seniors might experience poor healthcare services at home because of the shortage of medical devices and the lack of proper support and healthcare systems [12]. Moreover, the health system in Iran may not be able to provide efficient, necessary, and durable healthcare services to a large number of senior citizens. Consequently, they are more likely to receive long-term healthcare services from family caregivers. In addition, there is a lack of long-standing health insurance in Iran [13]. Throughout the caregiving process, caregivers may experience stress and burden due to vigorous care activities; it can negatively affect their physical, mental, and social lives that lead to the reduction of their quality of life [14]. A meta-analysis of the physical and mental health effects of caregiving has concluded that there is a higher level of depression and physical health problems among caregivers compared to non-caregivers [15]. Irfan et al. found that about 46% of participants reported adverse impact of caregiving on their lives. Moreover, there was a negative relationship between daily activities such as sleeping, eating, as well as exercising, and care in the majority of participants (65%). In addition, providing care could have undesirable impacts on physical (40.8%), psychological (47.8%), family (48.5%), occupational (51.3%), and social (53%) aspects of life [16].

There is a difference between caregiving burden and family caregiver’s challenges. The burden on caregivers refers to complicated reactions to caregiving experience-induced stressors such as emotional, physical, social, financial, and psychological factors [17]. On the other hand, the overwhelming and anxiety-provoking challenges (e.g., sleeplessness, isolation, time management, financial issues, hopelessness, stress, and losing privacy) while taking care of the patients at home are likely to pressure family caregivers [18]. Consequently, the caregiving burden may reflect the inappropriate decisions to resolve such challenges [19]. Even though the caregiving burden has been investigated widely pertinent to senior citizens with MCCs, there is scarce research on family caregivers’ challenges in the literature.

Some studies have been conducted on the experiences of family caregivers supporting seniors with MCCs. In order to explore the definition of providing care for patients with MCCs, Peacock et al. [20] found that family caregivers are faced with life-on-hold challenges due to the demand for care, feeling of isolation, and making all decisions regarding the management of older residents’ diseases [20]. Kuluski et al. [21] also examined patient care objectives from different perspectives, such as seniors with MCCs, their family caregivers, and family physicians. In general, common objectives were to maintain patients' functional independence and manage their functional symptoms or challenges [21]. Giovannetti et al. [22] indicated that increasing health care task difficulty is associated with an increase in stress and depression among caregivers of seniors with MCCs [22]. Moreover, Ploeg et al. [4] investigated the experience of managing MCCs in the community from the seniors, family caregivers, and health care providers’ perspective reflecting difficult, tedious, and complicated care; planning medication and physician appointments; being split into pieces; adhering to physicians’ prescription; relying on family members and friends; as well as difficulty in accessing outside support [4]. Williams et al. [8] concluded that providing care had an impact on caregivers' work, family, and health. Participants claimed that it was difficult to manage a job with the right to care and respective tasks. They have experienced a loss of intimacy and growing family conflicts due to the demand for care; in addition, their health status declined because of the physical and emotional demands of providing care for seniors with MCCs. These researchers asserted that age, ethnicity, employment status, geography, social interactions, and immigration status could affect life care experience [8]. Finally, Gill et al. [23] concluded that caregivers had reported various challenges regarding the system (long waiting time, poor communication, & poor treatment coordination) and patients (care management). They were also faced with challenging decision-making while providing medical care to seniors with MCCs [23].

All of these studies have been conducted in the Canadian context. Most of these studies focused on caregivers’ challenges in managing seniors with MCCs. It might be unlikely to generalize the findings of previous studies to the Iranian community given that the family caregivers of the older residents would confront various challenges that are sometimes culture-specific and situation-specific. Some studies have highlighted the caregiving burden on family caregivers for such patients; nonetheless, the present study aims to investigate the influence of this burden on different dimensions of their lives. Hence, the following research question is proposed: “What challenges do family caregivers of senior citizens with MCCs experience concerning different dimensions of their lives?” Although the analysis of the experience of providing care for seniors with MCCs and its impact on the daily life of family caregivers requires the implementation of a qualitative method, the literature review indicates that it has been disregarded in this context.

Meanwhile, the existing body of research in developing countries has explored the caregiving situation where the older person is dealing with a single problem, not multiple problems. There is no study on the experiences of family caregivers of seniors with MCCs in developing countries such as Iran; thus, it is imperative to conduct a qualitative study. The findings of the present study can provide important information to facilitate the development of innovative interventions based on caregivers’ challenges using a caregiver-centered approach.

## Methods

### Study design

This study was conducted using the conventional content analysis (CCA) approach, which facilitates obtaining participants’ information directly; hence, no predetermined attitudes are required. The results of the content analysis are primarily derived from the participants’ distinctive and authentic perceptions [24]. In this way, CCA is a suitable approach to explain the challenges imposed to family caregivers of seniors with several chronic diseases in Iran.

### Setting and sample

The present study was conducted in Khorramabad, Iran, in 2019–2020. Participants included 13 family caregivers of seniors with MCCs who were selected based on purposive sampling from two health centers and outpatient clinics of two state hospitals in Khorramabad. For this purpose, the following inclusion criteria were taken into account: 1- Family caregivers of seniors with multiple chronic diseases who need constant care by their family members; 2- Willingness to participate in the study; 3- Having at least three months of experience in patient care; 4- Being able to communicate, and; 5- Having no history of severe physical illness or known mental disorders. Besides, participants were allowed to leave the study at any stage.

Having obtained necessary permissions, the researcher referred to the health centers to select the study participants. At first, the objectives of the study were explained. Then, the electronic health profiles, including the list of the senior citizens with MCCs (their name, contact number, and the type of the disease), were reviewed for eligible participants. The researcher also had access to their family caregiver’s contact information, and these caregivers were contacted to ensure the older resident were currently receiving caregiving services at home. Since the present study requires a minimum of three months of home caregiving as the inclusion criteria for the caregivers, the family caregivers were asked about the duration of the older patients’ care needs. After obtaining written informed consent, the eligible caregivers were interviewed. It is also noteworthy that the majority of caregivers from the health centers entered the study, along with three more participants from an outpatient healthcare center.

### Ethical consideration

The proposal of this study was reviewed and approved by the Ethics Committee of Tarbiat Modares University (no: IR.MODARES.REC.1398.045). After obtaining the required permission from the Ethics Committee, the interviews were conducted. Participants were informed about the objectives and significance of the study, and then informed consent was obtained to record the interviews. They were also ensured about the confidentiality of data and their right to leave the study at any moment.

### Data collection and procedure

Semi-structured interviews were used to collect the data. Interviews were conducted individually in the caregiver’s house or workplace, and were recorded using a tape recorder. The interview would begin with the following questions: "Explain the experience of the daily care for your patient?", "Has it affected your daily life routines?", and "What problems have you experienced due to providing care?". Then, the researchers would ask follow-up questions based on participants' answers, such as "Please explain more", "What did you mean? ", or "Give an example". Eventually, they were asked to comment on any remaining issues. The interview would take an average of 40 min. Interviews continued until data saturation was reached. Consequently, data saturation was obtained after the tenth interview in the present study, but three more interviews were conducted to ensure the adequacy of sampling.

### Data analysis

Units of analysis contained each transcript of the interviews. Then, each interview was reviewed several times according to the model proposed by Elo and Kyngäs [25], and then data analysis was performed using an inductive approach after the data made sense. Important paragraphs were highlighted and summarized through reading line by line and were divided into meaning units. Each meaning unit was assigned a code. In the next step, the codes indicating a particular subject were integrated into a sub-category. Finally, the main categories were formed by comparing the subcategories.

### Rigor of research

The proposed criteria by Guba and Lincoln [26] were used to obtain the trustworthiness of the data. In order to ensure the credibility of data collection, the researcher was entirely engaged with the data for a long time to perform constant analyses and comparisons. Then, incomprehensible sentences were resent to the participants for further clarification (member check). Dependability was achieved through quick transcription after reviewing the extracted interviews, codes, and categories by research team members. In order to confirm conformability, peer check and external check were used to ensure if they had similar interpretations from the extracted results. It is noteworthy that the researcher attempted to disregard his assumptions in the process of data collection and analysis. Transferability of data was sought through diversity in sampling as well as the demonstration of participants' characteristics, method of data collection, and analysis; it can also help other scholars follow the research procedure.

## Results

In the present study, most of the caregivers were female (*n* = 10), and 6 participants were seniors’ daughters. Most of them (*n* = 9) provided care for over 12 h a day. All the family caregivers, except one case, reported more than three years of constant care for seniors (Table 1). Most of the older residents were male patients aged between 61 and 84 years (Table 2). The analysis of the collected data led to a total of 263 primary codes; however, after reviewing and removing duplicated or overlapping codes using multiple reductions, 98 primary codes remained. Then, they were classified into 18 subcategories and 6 main categories, including the impact of caregiving on family relationships, disruption of social relationships, disruption of personal and occupational plans, physical health-related issues, negative emotions, and dealing with high costs of care (Table 3).

**Table 1:** Characteristics of family caregivers

Participant	Demographic characteristics												
	Sex	Age	Marriage	Education	Residence	Occupation	Relation	Disease	Economic status	Care time	Duration of care	Help from others	Taking care of others (patients)
P1	Male	50	Married	Graduate	Urban	Employee	Son	No	Average	Over 12 h	Over 3 years	Yes	Yes
P2	Female	51	Married	Undergraduate	Urban	Household	Daughter-in-law	Yes	Low	Over 12 h	Over 3 years	No	No
P3	Female	50	Married	Illiterate	Urban	Household	Daughter-in-law	Yes	Low	Below 6 h	Over 3 years	No	No
P4	Female	56	Married	Illiterate	Urban	Household	Spouse	No	Average	Over 12 h	Over 3 years	No	Yes
P5	Female	39	Single	Graduate	Urban	Jobless	Daughter	Yes	Average	Over 12 h	Over 3 years	No	No
P6	Male	38	Single	Graduate	Urban	Self-employed	Son	Yes	High	Over 12 h	Over 3 years	No	Yes
P7	Female	39	Single	Graduate	Urban	Self-employed	Daughter	No	Average	6 – 12 h	Over 3 years	No	No
P8	Female	51	Married	Undergraduate	Urban	Household	Daughter	No	Average	Over 12 h	Over 3 years	Yes	Yes
P9	Female	60	Married	Illiterate	Urban	Household	Spouse	Yes	Average	Over 12 h	1 to 3 year	No	No
P10	Female	39	Divorced	Graduate	Urban	Jobless	Daughter	No	Average	6 – 12 h	Over 3 years	No	No
P11	Male	52	Married	Graduate	Urban	Employee	Son	Yes	Average	Below 6 h	Over 3 years	Yes	No
P12	Female	41	Married	Graduate	Urban	Employee	Daughter	No	Good	Over 12 h	Over 3 years	No	No
P13	Female	35	Married	Graduate	Urban	Employee	Daughter	No	Good	Over 12 h	Over 3 years	Yes	No

**Table 2:** Characteristics of older adults

Older adults	Demographic characteristics										
	Sex	Age	Marriage	Education	Residence	Occupation	Hospitalization during the past 3 months	Family life	The number of chronic diseases	Diagnosis with chronic diseases	Chronic diseases
OA1	Female	82	Widowed	Illiterate	Urban	Household	Yes	With children	2	1 to 5 year	Kidney disease and bone marrow cancer
OA2	Female	80	Widowed	Illiterate	Urban	Household	No	With children	3 or more	Over 10 years	Diabetes, hypertension, and stroke
OA3	Female	74	Widowed	Illiterate	Urban	Household	Yes	With children	2	Over 10 years	Diabetes and hypertension
OA4	Male	66	Married	Undergraduate	Urban	Retired	No	With wife and children	3 or more	Over 10 years	Diabetes, hypertension, and cardiovascular disease
OA5	Female	62	Widowed	Illiterate	Urban	Household	Yes	With children	3 or more	Over 10 years	Diabetes, hypertension, cardiovascular, kidney, and pulmonary disease
OA6	Male	62	Married	Diploma	Urban	Retired	Yes	With wife and children	3 or more	Over 10 years	Diabetes, hypertension, cardiovascular and kidney disease
OA7	Female	62	Widowed	Undergraduate	Urban	Household	Yes	With children	3 or more	Over 10 years	Diabetes, hypertension, cardiovascular disease
OA8	Male	84	Married	Undergraduate	Urban	Retired	No	With wife	3 or more	1 to 5 year	Cardiovascular disease, Alzheimer, and Parkinson
OA9	Male	71	Married	Illiterate	Urban	Retired	Yes	With wife and children	3 or more	1 to 5 year	Diabetes, hypertension, cardiovascular and kidney disease
OA10	Male	67	Married	Illiterate	Urban	Retired	No	With wife and children	3 or more	Over 10 years	Diabetes, cardiovascular disease, hepatitis, Parkinson
OA11	Female	68	Married	Illiterate	Urban	Household	Yes	With wife	2	Over 10 years	Diabetes and hypertension
OA12	Male	80	Married	Undergraduate	Urban	Retired	Yes	Alone	3 or more	1 to 5 year	Diabetes, hypertension, stroke, and gastric cancer
OA13	Male	61	Married	Diploma	Urban	Retired	Yes	With wife and children	2	1 to 5 year	Diabetes and hypertension

**Table 3:** Challenges in Iranian Family Caregivers of Older Adults with MCCs

main categories	Sub-categories
The impact of caregiving on family relationships	Declined conjugal relationship
	Ignoring their own children’s needs
	Children incompatibility
	Impaired emotional atmosphere
Disruption of social relationships	Limited activities and social interactions
	Declined contribution to community conventions and activities
Disruption of personal and occupational plans	Full-time engagement
	Care-Job conflicts
	Disregarding themselves
	Interference in routine life
Physical health-related issues	Changes in sleeping patterns due to caregiving
	Physical problems
Negative emotions	Being depressed
	Being anxious
	Disappointment with the seniors’ recovery
	Psychological distress
Dealing with the high costs of care	Costly medicine and equipment
	Costly professional care

### 1- The impact of caregiving on family relationships

Participants in this study had to spend much time with the seniors to provide care for them, which would often lead to overlooking their spouse and children. Accordingly, family caregivers may not have enough time or physical and mental energy to pay attention to their spouses and children. Providing care for the seniors has kept family members apart from each other, and there has been a lot of struggles and tensions among family members that has diminished life enjoyment.

The burdensome caregiving activities lead to weariness and boredom. Hence, family caregivers may spend less time proclaiming their love toward their spouses and engaging in recreational activities with their partners that threatens their conjugal life and develops incompatibilities accordingly. Consequently, married caregivers are likely to experience cold and inefficient marital relationships. Furthermore, taking care of older residents at home results in continuous struggles as well as physical and emotional failure in married life. It may also impose strict limitations in spending time with children and meeting their immediate needs (e.g., medical, emotional, and educational needs), which can exacerbate the fights and disputes at home and create anger and anxiety in the family.

"This new situation has affected my relationship with my husband, but you cannot complain. The conjugal relationship declines …., we cannot spend enough time together." (Participant No. 3, the senior’s daughter-in-law)

"We usually disregard our children since my wife and I are busy taking care of my mother". (Participant No. 1, the senior’s son)

"My daughter sometimes gets angry and argues with us because we have to stay home most of the time; even though we know she is often right, we decide to ignore her wishes to control her expectations". (Participant No. 2, the senior’s daughter-in-law)

"The home atmosphere is not happy … there is much negative energy … and my family constantly complain to me because I am the only one who took on this responsibility" (Participant No. 2, the senior’s daughter-in-law)

### 2- Disruption of social relationships

Humans are social creatures who wish to connect to other people as well as organizations in the community. Individuals need to build relationships with others. For instance, they would tend to attend social, sports, and science events, take part in family and friends gatherings, and communicate with peers. Caregivers have stated that the process of providing care for the seniors has reduced social contact with friends and relatives due to imposing time (time-consuming care) and location constraints (forced to stay with the senior citizens); in addition, this reduction has led to the exclusion of social activities such as tourism, pilgrimage, and education. A large number of family caregivers go to work, and they cannot leave their job because of the high expenses of caregiving procedures. As a result, they need to spend most of their time either at work or at the older person’s bedside, and they may have very limited time for social events. Taking care of the senior citizens with MCCs imposes a more significant burden on family caregivers because these patients are mostly incapable of performing their daily life activities by themselves. Consequently, the amount of pressure introduced by the long and tiring hours of caregiving will substantially affect and restrict family caregivers’ social lives.

"My relationship with my relatives and friends has decreased significantly. They usually go out, but I can only have a distant relationship with them. However, I should say that they sometimes visit me.” (Participant No. 10, the senior’s daughter)

"I want to go on a pilgrimage to Karbala, or I want to take a trip with my family, but I cannot leave home". (Participant No. 2, the senior’s daughter-in-law)

"Taking care of my father was a big obstacle for me’ I could not continue my education" (Participant No. 13, the senior’s girl)

### 3- Disruption of personal and occupational plans

People have various goals in their personal lives and plan to accomplish such goals concerning the priorities; nevertheless, taking care of the seniors leads to the prioritization of seniors’ needs over their own goals. Caregivers asserted that accepting the role of a caregiver has affected their personal and occupational goals and plans. The wide range of tasks requires caregivers to spend most of their valuable daily time providing care. In other words, older residents with MCCs may seek constant support, and it is relatively impossible for family caregivers to leave them alone. Hence, they need to prioritize the patient’s demands over their own needs and lives. Family caregivers should plan their daily or weekly schedule according to the older person’s healthcare demands, which results in ignoring personal desires and plans for an extended period. Moreover, employed caregivers had to reduce their working hours and limit their occupational objectives to take care of the seniors efficiently. Besides, discrepancies in occupational duties and the lack of focus while performing work-related tasks may inevitably cause career drawbacks.

"I have to wake up at seven in the morning to inject insulin to my husband. I should give him the medicine, change his clothes, prepare food, and inject insulin again. This is a routine day for me … and I can no longer do my own chores". (Participant No. 9, the senior’s spouse)

"I am worried about my parents. I am 38 years old, and I am not married. I imagine if I get married, I may not be able to take care of them". (Participant No. 6, the senior’s son)

"I have to adapt to this situation … I have to adjust my schedule so that I can take care of my father's affairs". (Participant No. 10, the senior’s daughter)

"Before my parents' illness, I used to have several international business trips. But I have to cancel all my trips abroad because I need to take care of them". (Participant No. 6, the senior’s son)

### 4- Physical health-related issues

It is crucial to have enough and regular rest to promote physical health. Family caregivers are forced to disturb their regular sleeping routine because the seniors sometimes need help during the night. Sometimes, caregivers cannot sleep well due to the fear of any possible problems for the seniors, leading to daily weariness and exhaustion. Participants in the present study claimed that the seniors need to be relocated for health care and treatments, which has caused chronic pain in muscles, joints and, bones. Caregivers have also experienced other physical problems such as physical fatigue, headache, and loss of weight. Family caregivers have primarily indicated physical tension and fatigue, which is aggravated because older residents with MCCs are not capable of performing daily life activities like cleaning, cooking, and moving inside the house. Therefore, caregivers should be crucially involved with providing support and care, even at the expense of creating further physical issues for themselves.

"If my father feels really sick, I will be worried about him, and I cannot sleep". (Participant No. 10, the senior’s daughter)

"Since my husband is forced to use diapers, he is not able to walk, and it is challenging for me to move him because I have backache too. I have hurt my pelvis because I should move him." (Participant No. 9, the senior’s spouse)

### 5- Negative emotions

It is substantially difficult for family caregivers to witness the older person’s struggles and discomfort. Given the tremendous pressure of providing care for the seniors, family caregivers would feel indifferent to life and helpless; besides, they would suffer from loss of mood and sadness, which indicates a level of depression. Family caregivers are constantly anxious about the spread of the disease or the occurrence of a new incidence that might lead to the death of the older adult or aggravation of their disease. Several family caregivers consider some of the interventions (changing their clothes, using the catheter, and taking care of the vomit container) unpleasant and disgusting. Despite family caregivers’ great efforts in providing care to the seniors, they are disappointed about the improvement and recovery of the patient because the seniors suffer from several chronic diseases. Family caregivers also expressed their feelings about the caregiving experience. They complained that they had been offended by the lack of appreciation from others, the accusation of providing inadequate care, particularly with the aggravation of the seniors’ condition, and being abused. Some caregivers stated that they would often become angry with the seniors, and they would feel guilty and remorseful accordingly. Moreover, family caregivers are willing to give priority to the older resident, which may endanger their own physical and emotional health. The imposed distress, weariness, and despair feelings are likely to have significant detrimental impacts on caregivers.

"I feel really tired … our mental state is affected by this issue … sometimes, I feel I cannot tolerate the condition anymore …. I often lose my patience … when the senior’s condition becomes worse, it makes you angry and anxious … it is really tough". (Participant No. 5, the senior’s daughter)

"We are constantly under pressure because we do not know what is going to happen next … when I want to go to the bathroom, I usually look at my father and his chest to check if he is breathing. We would always screen his conditions. When he remains silent, I would approach him and monitor his conditions" (Participant No. 10, the senior’s daughter)

"We have been treating his heart for several years, but there has been no improvement. We constantly attempt to control his blood sugar, but it is always abnormal; hence, he needs to take insulin. It is impossible to recover from Parkinson. Besides, hepatitis might contribute to various diseases. What are just looking for a miracle". (Participant No. 10, the senior’s daughter)

"Other siblings no longer appreciate my efforts". (Participant No. 2, the senior’s daughter-in-law)

"Sometimes I get nervous; however, I usually feel ashamed and I apologize afterward". (Participant No. 10, the senior’s daughter)

"My brothers and sister live in another city and are busy with their own lives. I am the only one who takes care of our father. It is a bad feeling. I think I am being abused." (Participant No. 7, the senior’s daughter)

### 6- Dealing with the high costs of care

Caregivers argued that, regardless of the imposed problems in their personal, family, social, physical, and psychological life, they are also faced with financial issues. The majority of such financial difficulty is associated with various treatment expenses, including medicine and necessary equipment such as diapers, disposable catheters, dressings, serum, medication injection, as well as the expenses regarding some specialized care, including a home visit by the physician, nurse, or physiotherapist. Given that it requires a significant amount of money to cover the taking care of older citizens with MCCs and the majority of such patients do not have a paid job, family caregivers are constantly seeking financial resources. These caregivers often decide to restrict their personal and family budgets to cover the caregiving costs. Furthermore, family caregivers in Iran are usually forced to pay out-of-pocket to access immediate healthcare services. Finally, it is noteworthy that the growing inflation rate in Iran can also aggravate the existing financial burden on the family caregivers.

"We have to buy much equipment because they are frequently consumed; for instance, we are always out of diapers because we need to change his diapers three times a day … besides, we are in constant need of plaster and dressings, catheters, serum, and other equipment". (Participant No. 1, the senior’s son)

"We asked for home-visit physiotherapy services due to my mother's condition. The physiotherapist visited my mother for two sessions, but the expenses are really high". (Participant No. 1, the senior’s son)

## Discussion

This study aimed to identify the challenges that affect family caregivers of seniors with multiple chronic diseases. " The impact of caregiving on family relationships " is regarded as one of the major challenges. Family caregivers have to spend much time with the seniors. Hence, they fail to pay enough attention to their spouse and children. Caregivers prioritize the measures they should take for their patients [27]. This may lead to incompatibility between the children and the caregiver due to lack of attention; besides, the caregiver will experience a cold relationship with his/her spouse because of the lack of proper emotional connection. Williams et al. (8) reported that providing care for seniors with MCCs influences caregivers' family relationships, including conjugal relationships [8]. It is also likely to observe adverse social effects in family relationships with spouses, children, and other close people. Nonetheless, this issue has received relatively little attention in the literature [28]. The caregiving burden can cause frustration among family members and disrupts the emotional atmosphere at home. Amirkhanyan and Wolf [29] concluded that the psychological effects of providing care are experienced by all family members [29]. In the present study, the following sub-categories were extracted as novel findings regarding family caregivers of seniors with MCCs: "Ignoring their own children’s needs", "children incompatibility", and "Impaired emotional atmosphere". Therefore, it is necessary to consider these three subcategories for the effectiveness of caregiver-related interventions.

As another challenge for family caregivers, "disruption of social relationships" can be contributed to place and time limitations. These restrictions are imposed due to the seniors’ condition, who are often unable to relocate and do their daily activities. Ploeg et al. [6] claimed that the multifaceted nature of caregiving responsibilities in some caregivers of seniors with MCCs and Alzheimer’s could result in constant homestay [6]. This situation leads to the deprivation of social activities such as education as well as reduced interactions with friends and travels. Blusi et al.  [30] indicated that the intensity of caregiving tasks and continuous residence at home might lead to feeling social isolation among caregivers [30]. To reduce such isolation, caregivers should be engaged in virtual associations and respite care. It is also noteworthy that previous studies have not examined dropping out of school; hence, the present study is the first one in this case.

Family caregivers claimed that it could also lead to the Disruption of personal and occupational plans. Caregivers are faced with two fundamental responsibilities: their personal life and taking care of the seniors. The majority of caregivers prefer the seniors’ affairs over their personal life, which results in the disruption of everyday life routines. It is believed that seniors with MCCs have laborious and time-consuming needs that are often more important than the caregiver’s personal needs [6]. Peacock et al. [20] used the term “life on hold” for caregivers since they were also forced to make adjustments in their jobs (e.g., leave of absence, relocation, & even leaving work) [20]. Faronbi et al. [31] asserted that taking care of the seniors prevents caregivers from attending their daily or family occasions and also other personal responsibilities; in addition, they are required to spend less time, leave, or disregard their family and work [31]. Therefore, it is suggested to strike a balance between personal life and care to be able to manage both sides properly [32, 33].

Physical health-related issues while taking care of the seniors are regarded as another challenge. Caregivers are forced to move the seniors for different tasks, which can cause Physical issues such as backache, painful wrist, pelvic pressure, pain in the body, and bruises for the caregiver. The findings of Alam & Abd Elhameed’s study revealed that caregivers' physical health status has reduced. They argued that caregivers who take care of patients with chronic illnesses were at greater risk of losing physical strength and deteriorating their health [34]. There is no definite timetable for such care, and this can disrupt the caregiver's sleeping schedule as well. It leads to several problems such as nocturnal sleeping disorders, insomnia, irregular sleeping schedule, and being forced to wake up early in the morning. Sleeping disorders refer to a common and complex problem among the family caregiver population [35]. The physical health status of family caregivers is a crucial health issue that requires serious attention from health care providers; hence, it is imperative to take into account some strategies to promote their health as a priority in health care in the community.

Caregivers also reported Negative emotions as another challenge. Family caregivers become indifferent, unmotivated, and helpless over time; they also suffer from grief and depression as a result of constant stress because they are worried about losing the seniors or the exacerbation of their illnesses. The findings of three meta-analyses estimated that 26% to 57% of caregivers might experience depression accordingly [36–38]. Ploeg et al. [39] conducted a seminal study and concluded that taking care of a person with dementia and MCCs is associated with significant emotional and psychological burdens [39]. Despite the great efforts by family caregivers, they are often hopeless regarding the seniors' recovery because they are suffering from multiple illnesses. Moreover, caregivers argue that they are sometimes offended by the lack of gratitude from others, the accusation of inadequate care, and the fact that they are being abused. The review of the related literature demonstrated that the disappointment with the improvement of seniors with MCCs and their psychological distress have not been addressed in previous studies, and therefore they can be considered unique findings of this study.

Caregivers may face dealing with the high costs of care as well. Undoubtedly, providing care requires specialized equipment and assistance that imposes significant expenses on the caregiver and the family. As a result, there might be a reduction in their share of preparing the necessities of life. In Iran, insurance companies do not usually cover healthcare expenses, including medicine, equipment, and specialized assistance. Therefore, increased health care costs and insufficient insurance coverage have led to a potentially devastating financial burden on many families [40]. Williams et al. [8] reported that financial health emerged as a significant component of sustainable care among caregivers of seniors with MCCs, provided that financial problems would add to the treatment and care burden [8]. Therefore, it seems necessary that insurance policies, charitable donations, and governmental grants should be highlighted to reduce the financial hardship of caregivers. Furthermore, dealing with the high costs of care is divided into "costly medicine and equipment" and "Costly professional care" in the present study. This categorization has not been proposed in previous studies where it can contribute to public policymaking in the future.

Given the tiring and stressful nature of providing care for older people with MCCs, nurses should attempt to identify the family caregivers’ potential problems through interactions, propose necessary instructions, and provide sufficient support to help them overcome such problems and deliver efficient care to these patients. According to the findings of the present study, family caregivers of patients with MCCs have sought more crucial support and attention. Consequently, it is essential to establish more healthcare centers, improve insurance systems, as well as design and implement effective counseling measures for this group of caregivers.

### Limitations

The present study also had its limitation. Due to the limited number of family caregivers, it is not possible to generalize the findings to other family caregivers’ populations. Moreover, participants’ attempts to please the researcher (participant bias) can be another limitation of the study. This study represents the experiences of purposively selected family caregivers of two health centers and outpatient clinics of two public hospitals in Khorramabad, Iran. Also, due to the small number of male caregivers in these centers, we could not include more male caregivers in our study.

## Conclusions

The findings revealed that providing care for seniors with MCCs can influence different aspects of the family caregivers’ life, such as their family and social relationships. Besides, their personal and occupational plans will be interrupted accordingly. On the other hand, the caregiver might be susceptible to physical health-related issues, negative emotions, and dealing with the high costs of care. Therefore, it is crucial to design a system so that caregivers can manage the impact of this situation on their lives. Therefore, it is suggested that interventional research should be conducted to discover practical approaches to support family caregivers.

### Implications

Healthcare providers should design and plan interventions based on a caregiver-centered approach to help overcome caregivers’ challenges. Results of the present study can be used as a basis for developing interventional programs to address family caregivers’ challenges. These findings can also be considered as general indicators for public policies so as to support family caregivers; for instance, the findings revealed that dealing with the high cost of care might be very useful in determining public policies for insurance and governmental support. It is assumed burdensome to take care of the senior citizen with multiple chronic diseases. Family caregivers are prone to different psychological and bodily issues; thus, the severe mental and physical overtiredness of taking care of the older members of the family may result in providing inefficient healthcare services. In other words, family caregivers’ exhaustion may lead to durable and detrimental impacts on older patients. Hence, the healthcare systems should be responsible for meticulous analysis of the family caregivers’ conditions and should propose immediate solutions for their problems, particularly if they are taking care of the senior citizens at home.

## Data Availability

The data of this study include the face-to-face interview transcripts. Raw data cannot be publicly available due to the risk of compromising participant privacy. However, data are available from the corresponding author on reasonable request.

## Abbreviations

MCCs: Multiple chronic conditions
CCA: Conventional content analysis
OA: Older adults
